# Barriers and facilitators to dietary adherence in adults with chronic kidney disease: a protocol for systematic review

**DOI:** 10.1186/s13643-026-03080-6

**Published:** 2026-03-26

**Authors:** Ravi Shankar, Anjali Bundele, Joshua Low, Wei-Zhen Hong

**Affiliations:** 1 Clinical Research & Innovation Office, NHG Health, Tan Tock Seng Hospital, Singapore, Singapore; 2 Fast and Chronic Programmes, Alexandra Hospital, Singapore, Singapore; 3 Yong Loo Lin School of Medicine, National University of Singapore, Singapore, Singapore; 4 Division of Nephrology, Department of Medicine, National University Hospital, Singapore, Singapore; 5 Department of Pharmacy, Alexandra Hospital, Singapore, Singapore

**Keywords:** Chronic kidney disease, Dietary adherence, Barriers, Facilitators, Theoretical Domains Framework

## Abstract

**Background:**

Dietary adherence is a critical component of managing chronic kidney disease (CKD) and its associated complications. However, patients with CKD often struggle to adhere to dietary recommendations due to various barriers and facilitators that influence their behavior. Existing reviews on this topic have been limited in scope or have not used a theoretical framework to guide the analysis. This systematic review aims to identify and synthesize the evidence on barriers and facilitators to dietary adherence in CKD patients using the Theoretical Domains Framework (TDF) to categorize the identified factors. Using a PICo framework: Population—adults with chronic kidney disease (all stages); Phenomena of Interest—barriers and facilitators to dietary adherence; Context—any healthcare setting globally; Outcomes—TDF-categorized factors influencing dietary adherence and their associations with adherence measures.

**Methods:**

We will conduct a comprehensive search of electronic databases (PubMed, Web of Science, Embase, CINAHL, MEDLINE, The Cochrane Library, PsycINFO, and Scopus), grey literature, and reference lists of included studies to identify quantitative, qualitative, and mixed-methods studies that report on barriers and facilitators to dietary adherence in adult CKD patients. Two independent reviewers will screen studies for inclusion, extract data, and assess the risk of bias using established tools (NOS, CASP, MMAT, RoB 2). We will use a narrative synthesis approach guided by the TDF to categorize and synthesize the findings. If appropriate, meta-analyses will be conducted for specific outcomes or associations. Potential meta-analyses may examine associations between specific barriers/facilitators and dietary adherence measures, biochemical outcomes (e.g., serum phosphorus, potassium levels), or clinical outcomes where sufficient homogeneous data are available. The GRADE and GRADE-CERQual approaches will be used to assess the overall quality of evidence.

**Discussion:**

This systematic review will provide a comprehensive and theoretically informed synthesis of the barriers and facilitators to dietary adherence in CKD patients. The findings will inform the development of targeted interventions, patient education strategies, and future research priorities in the field of CKD dietary management. By using the TDF to guide the analysis, we will provide a structured understanding of the complex factors influencing dietary adherence, which can inform the design of theory-based interventions to improve patient outcomes.

**Systematic review registration:**

PROSPERO CRD42024571389

**Supplementary Information:**

The online version contains supplementary material available at 10.1186/s13643-026-03080-6.

## Introduction

Chronic kidney disease (CKD) is a progressive condition characterized by a gradual loss of kidney function over time. It affects an estimated 10% of the global population and is associated with significant morbidity, mortality, and healthcare costs [1]. As kidney function declines, patients face numerous challenges, including the management of comorbidities, adherence to complex medication regimens, and the need for significant dietary modifications [2].

Dietary management is a cornerstone of CKD treatment, playing a crucial role in slowing disease progression, managing complications, and improving quality of life [3]. Depending on the stage of CKD and individual patient factors, dietary recommendations may include restrictions in protein, sodium, potassium, phosphorus, and fluid intake, as well as careful monitoring of overall caloric intake [4]. These dietary modifications are often complex and can significantly impact patients’ daily lives and social interactions.

Despite the importance of dietary management in CKD, adherence to prescribed dietary regimens remains a significant challenge for many patients. Studies have reported adherence rates ranging from 20 to 70%, depending on the specific dietary component and the method of assessment [5]. Adherence rates vary significantly across CKD stages, with pre-dialysis patients often showing different adherence patterns compared to those on dialysis or post-transplantation. Patients on hemodialysis may face unique challenges with fluid and phosphorus restrictions, while peritoneal dialysis patients may have different protein requirements. It is important to note that adherence assessment methods vary considerably in their reliability and validity. While serum markers (such as phosphorus, potassium levels, or interdialytic weight gain) are commonly used as proxy measures for dietary adherence, these may not accurately reflect adherence due to multiple biological factors including residual kidney function, medication effects, bone disease, and individual metabolic variations. Patient-reported measures, dietary recalls, and behavioral assessments may provide more direct insights into actual dietary behaviors, though each has its own limitations. Poor dietary adherence can lead to various adverse outcomes, including accelerated disease progression, increased risk of complications, and reduced quality of life [6].

Understanding the factors that influence dietary adherence in CKD patients is crucial for developing effective strategies to support patients in managing their diet. These factors can be broadly categorized into barriers (factors that hinder adherence) and facilitators (factors that promote adherence). Previous research has identified various potential factors, including patient-related (e.g., knowledge, beliefs, self-efficacy), social (e.g., family support, cultural factors), healthcare system-related (e.g., access to dietitian services, quality of patient education), and disease-related factors (e.g., comorbidities, symptom burden) [7, 8].

The Theoretical Domains Framework (TDF) is a comprehensive framework that synthesizes a wide range of behavior change theories into a single framework [9]. It consists of 14 domains that capture the potential influences on behavior, including knowledge, skills, social/professional role and identity, beliefs about capabilities, optimism, beliefs about consequences, reinforcement, intentions, goals, memory, attention, and decision processes, environmental context and resources, social influences, and emotion [10]. The TDF has been widely used to identify barriers and facilitators to healthcare professionals’ behavior change [11] and has recently been applied to understanding patient behavior, including treatment adherence [12, 13].

Applying the TDF to the context of dietary adherence in CKD patients can provide a structured approach to identifying and categorizing the complex factors that influence adherence. By mapping the identified barriers and facilitators to the TDF domains, we can gain a more comprehensive understanding of the key influences on dietary adherence and inform the development of theory-informed interventions to improve adherence.

Several reviews have examined aspects of dietary adherence in CKD patients, but none have comprehensively synthesized quantitative and qualitative evidence on barriers and facilitators across all CKD stages using a theoretical framework. Lambert et al. [14] focused on health literacy as a single potential barrier, while Desroches et al. [15] included various chronic conditions in their Cochrane review of adherence interventions. Other reviews have addressed specific dietary components such as adherence to phosphate control [16, 17] or overall treatment adherence [18]. To our knowledge, no review has comprehensively synthesized and theoretically categorized the barriers and facilitators to dietary adherence across all stages of CKD.

## Study objectives

The primary objective of this systematic review is to identify and synthesize the evidence on barriers and facilitators to diet adherence in patients with chronic kidney disease, using the TDF to categorize the identified factors. To clarify our approach, we will include all relevant studies reporting barriers and facilitators to dietary adherence, regardless of whether they explicitly use the TDF or any theoretical framework. During the synthesis phase, the review authors will map all identified barriers and facilitators to the appropriate TDF domains based on the descriptions and characteristics of these factors. This approach allows us to provide a comprehensive theoretical framework for understanding dietary adherence while including the full breadth of available evidence. Specific objectives include:

1. To identify and categorize barriers and facilitators to dietary adherence in CKD patients using the TDF domains
2. To explore variations in barriers and facilitators across CKD stages, dietary components, and patient populations
3. To assess the quality of existing evidence on barriers and facilitators to dietary adherence in CKD
4. To identify gaps in the current literature and provide recommendations for future research

The findings of this review will have several important implications. First, they will inform the development of targeted, theory-informed interventions to improve dietary adherence in CKD patients. By identifying key barriers and facilitators and mapping them to the TDF domains, healthcare providers and researchers can design more effective strategies to support patients in managing their diet. Second, the results will help in tailoring patient education and counselling approaches, allowing for more personalized and effective dietary management. Third, the review will highlight areas where further research is needed, guiding future studies in this field.

## Methods

This systematic review protocol is reported in accordance with the Preferred Reporting Items for Systematic Review and Meta-Analysis Protocols (PRISMA-P) guidelines [19]. The completed systematic review will be reported in accordance with the Preferred Reporting Items for Systematic Reviews and Meta-Analyses (PRISMA) guidelines [20]. The protocol has been registered with PROSPERO (registration number: CRD42024571389).

### Eligibility criteria

#### PICO framework

To clearly define the scope and focus of this systematic review, we have applied the PICo framework (Population, Phenomena of Interest, Context), which is more appropriate for qualitative and mixed-methods systematic reviews that do not focus on interventions [21]:

- Population
  Adult participants (≥ 18 years old) with chronic kidney disease of any stage (CKD stages 1–5, including those on dialysis and post-transplant).

- Phenomena of interest
  The barriers and facilitators to dietary adherence in adults with CKD. Barriers are defined as factors that hinder or negatively impact a person’s ability or willingness to adhere to dietary recommendations. Facilitators are factors that promote or positively influence dietary adherence.

- Context
  Studies conducted in any setting, including outpatient clinics, dialysis centres, hospitals, and community settings, and from any geographical location.

The primary outcomes of interest are the identified barriers and facilitators to dietary adherence in adults with CKD, categorized according to the TDF domains. Secondary outcomes may include measures of dietary adherence (if reported), associations between identified factors and dietary adherence, and patient-reported experiences and perspectives on dietary management.

By utilizing the PICo framework, we aim to provide a clear and focused approach to our systematic review, ensuring that we capture the relevant evidence to address our research question.

We will include both quantitative and qualitative primary research studies. Quantitative studies may include observational studies (cross-sectional, cohort, and case-control studies) and interventional studies (randomized controlled trials, quasi-experimental studies) that report on factors associated with dietary adherence. Qualitative studies may include those using methods such as interviews, focus groups, or ethnographic approaches to explore patients’ experiences and perspectives on dietary adherence. Mixed-methods studies will also be included if they provide relevant data on barriers or facilitators to dietary adherence.

#### Inclusion criteria

- Adult participants (≥ 18 years) with chronic kidney disease (any stage 1–5, including dialysis and post-transplant)
- Primary research studies (quantitative, qualitative, or mixed-methods)
- Studies reporting barriers and/or facilitators to dietary adherence or dietary management
- Studies examining factors associated with dietary behaviors in CKD
- Any geographical location
- Any healthcare setting

#### Exclusion criteria

- Pediatric populations (< 18 years)
- Studies focusing solely on medication adherence without dietary components
- Studies of acute kidney injury
- Animal studies or in vitro studies
- Reviews, opinion pieces, editorials, case reports, conference abstracts
- Studies where full text is not available after contacting authors

We will exclude review articles, opinion pieces, editorials, and conference abstracts. However, we will screen the reference lists of relevant review articles to identify additional primary studies.

We define dietary adherence as the extent to which a patient’s dietary behavior aligns with the recommendations provided by healthcare professionals. We acknowledge that studies may define and measure dietary adherence differently. We will include studies that report any measure of dietary adherence (objective, subjective, or proxy measures) and will extract and critically evaluate the adherence definitions used. Studies that identify factors associated with dietary behaviors, dietary knowledge, or dietary self-management without explicitly using the term ‘adherence’ will also be considered if the factors clearly relate to following dietary recommendations. This may include adherence to overall dietary recommendations or specific components such as protein, sodium, potassium, phosphorus, or fluid restrictions. Barriers are defined as any factors that hinder or negatively impact a patient's ability or willingness to adhere to dietary recommendations. Facilitators are factors that promote or positively influence dietary adherence.

Studies from any geographical location will be eligible for inclusion. We will include studies published in English from database inception to the date of our search. This broad time frame will allow us to capture the evolution of research in this field and identify any changes in barriers and facilitators over time.

### Information sources and search strategy

We will conduct a comprehensive search of electronic databases including PubMed (via NCBI), Embase (via Ovid), CINAHL (via EBSCOhost), MEDLINE (via Ovid), The Cochrane Library (via Wiley), PsycINFO (via Ovid), Scopus (via Elsevier), and Web of Science Core Collection (1945–present via Clarivate Analytics). In addition to these electronic databases, we will hand-search the reference lists of included studies and relevant review articles to identify any additional eligible studies. We will also search for grey literature through websites of relevant organizations (e.g., National Kidney Foundation, Kidney Disease: Improving Global Outcomes) and conference proceedings of major nephrology conferences (e.g., American Society of Nephrology Kidney Week, European Renal Association–European Dialysis and Transplant Association Congress) from the past 5 years. Conference proceedings will be searched to identify potentially relevant studies that may have been subsequently published as full papers. We will not include conference abstracts in the final review but will use them to identify researchers and studies for potential follow-up contact to locate full publications.

The search strategy has been developed in consultation with a medical librarian experienced in systematic review searching. We will use a combination of controlled vocabulary terms (such as MeSH terms in MEDLINE and PubMed, Emtree terms in Embase) and free-text keywords. For each controlled vocabulary term, we will also search corresponding keywords in title, abstract, and author keyword fields to ensure comprehensive coverage. The following search string will be used as a basis for our search strategy:

- (('kidney' OR 'renal' OR 'nephr*' OR CKD) AND ('diet*' OR 'nutrition' OR 'nutritional' OR 'food' OR 'eat*') AND ('adherence' OR 'compliance' OR 'self-management' OR 'engag*') AND ('barrier*' OR 'facilitator*' OR 'factor*' OR 'determinant*' OR 'challenge*' OR 'motivator*' OR 'cause*' OR 'relationship*' OR 'influence*' OR 'obstacle*' OR 'connection*' OR 'qualitative*')).

This search string will be adapted for each database (Appendix 1 in the supplementary file), taking into account database-specific indexing terms and syntax. The full search strategies for each database will be provided in an appendix to the final review.

### Study records and data extraction

We will use Covidence, a web-based software platform designed for systematic reviews, to manage the study selection process. All identified citations will be imported into Covidence, which will automatically remove duplicate records. Two independent reviewers will screen the titles and abstracts of all identified studies against the eligibility criteria. Studies that potentially meet the inclusion criteria will be retrieved in full text and independently assessed for eligibility by two reviewers. Any disagreements at either stage will be resolved through discussion, and if necessary, consultation with a third reviewer. We will document the reasons for the exclusion of full-text articles that do not meet the inclusion criteria. The entire selection process will be outlined in a PRISMA flow diagram in the final review.

A standardized, pre-piloted form will be used to extract data from the included studies for assessment of study quality and evidence synthesis (Appendix 2 in the supplementary file). Two reviewers will extract data independently; discrepancies will be identified and resolved through discussion, and a third reviewer will be consulted when necessary. We will contact the study authors to resolve any uncertainties. We will extract data on study characteristics (authors, publication year, study design, country of origin, setting, sample size, duration of follow-up), participant characteristics (age, gender, ethnicity, CKD stage, comorbidities, duration of CKD, renal replacement therapy status), dietary adherence (definition, assessment method, specific components, reported adherence rates), identified barriers and facilitators (including method of identification, strength of association, if reported, and TDF domain), additional outcomes (patient-reported experiences, other relevant findings), and analysis methods.

### Risk of bias assessment

The risk of bias assessment will be conducted independently by two reviewers, with disagreements resolved by discussion or consultation with a third reviewer if necessary. For quantitative studies, we will use the Newcastle–Ottawa Scale (NOS) to assess the quality of non-randomized studies in meta-analyses [22]. This tool evaluates studies based on three main categories: selection of study groups, comparability of groups, and ascertainment of exposure or outcome (Appendix 3 in the supplementary file). For randomized controlled trials, if any are included, we will use the revised Cochrane risk-of-bias tool for randomized trials (RoB 2) [23].

For qualitative studies, we will use the Critical Appraisal Skills Programme (CASP) Qualitative Checklist [24]. This tool assesses various aspects of qualitative research, including the appropriateness of the research design, recruitment strategy, data collection, relationship between the researcher and participants, ethical issues, data analysis, findings, and value of the research (Appendix 3 in the supplementary file). For mixed-methods studies, we will use the Mixed Methods Appraisal Tool (MMAT) [25], which allows for the assessment of qualitative, quantitative, and mixed-methods studies within a single tool.

The results of the quality assessment will be presented in tables and considered in the data synthesis. However, we will not exclude studies based on the quality assessment results, as even lower-quality studies may provide valuable insights into barriers and facilitators of dietary adherence.

### Deviations from protocol

Any deviations from this protocol during the conduct of the systematic review will be documented and reported in the final review with rationales for changes.

### Timeline

We anticipate completing this systematic review within 12 months of protocol publication, including: database searches (1 month), screening and selection (2 months), data extraction (2 months), quality assessment (1 month), synthesis and analysis (4 months), and manuscript preparation (2 months).

### Data synthesis

Given the expected heterogeneity in study designs, populations, and outcomes, we anticipate that a meta-analysis may not be appropriate. Instead, we plan to conduct a narrative synthesis of the findings from the included studies, guided by the framework proposed by Popay et al. [26].

The narrative synthesis will involve developing a preliminary synthesis, exploring relationships within and between studies, assessing the robustness of the synthesis, and developing a conceptual framework. We will tabulate the results of included studies, grouping them by study design, CKD stage, and type of factors investigated (barriers or facilitators). We will use this initial organization to identify patterns across studies. We will then examine factors that might explain differences in barriers and facilitators across different studies, such as CKD stage, cultural context, or healthcare setting, using conceptual mapping to visually represent these relationships. We will reflect on the strength of the evidence for each identified barrier and facilitator, considering the number and quality of studies supporting each finding. Based on the synthesized findings, we will develop a conceptual framework illustrating the interrelationships between various barriers and facilitators and their potential impact on dietary adherence in CKD patients.

We will categorize identified barriers and facilitators according to the 14 domains of the TDF (knowledge, skills, social/professional role and identity, beliefs about capabilities, optimism, beliefs about consequences, reinforcement, intentions, goals, memory, attention and decision processes, environmental context and resources, social influences, and emotion) [10]. This categorization will provide a structured understanding of the key influences on dietary adherence in CKD patients and inform the development of theory-informed interventions to improve adherence.

If sufficient data are available, we will conduct subgroup analyses to explore how barriers and facilitators may differ across CKD stages, specific dietary components, geographical regions or healthcare systems, and patient demographics. We will conduct sensitivity analyses to assess the impact of study quality on our findings by re-running our synthesis excluding studies deemed to be at high risk of bias. For narrative synthesis, subgroup analyses will involve separate synthesis and comparison of findings across different CKD stages, dietary components, geographical regions, and patient demographics. We will examine patterns and differences in reported barriers and facilitators across these subgroups through systematic tabulation and thematic comparison. Sensitivity analyses will involve re-examining the synthesis after excluding lower-quality studies to assess whether conclusions change, and examining the robustness of findings by considering only studies with stronger methodological quality or larger sample sizes.

If we identify a subset of studies with sufficiently homogeneous quantitative data, we will consider conducting meta-analyses for specific outcomes or associations. In such cases, we will use random-effects models to account for expected heterogeneity and assess statistical heterogeneity using the *I*^2^ statistic, with *I*^2^ values of 25%, 50%, and 75% considered as low, moderate, and high heterogeneity, respectively [27]. Potential summary measures for meta-analyses may include: odds ratios or risk ratios for associations between barriers/facilitators and adherence (for dichotomous outcomes); standardized mean differences for continuous adherence measures; correlation coefficients for relationships between factors and adherence scores. The choice of summary measure will depend on the outcome measurement scales and study designs of included studies.

For qualitative data, we will use thematic synthesis as described by Thomas and Harden [28]. This will involve line-by-line coding of findings, quotes, themes, and sub-themes from primary studies, organizing these codes into descriptive themes, and then generating analytical themes that go beyond the primary studies. The analytical themes will be mapped to the TDF domains to provide a comprehensive understanding of the barriers and facilitators to dietary adherence in CKD patients.

### Assessment of meta-biases and confidence in cumulative evidence

To assess potential reporting bias, we will examine whether studies that initially aimed to investigate barriers and facilitators to dietary adherence have remained unpublished. To identify potential unpublished studies and assess reporting bias, we will search PROSPERO for related systematic review protocols, ClinicalTrials.gov, and the WHO International Clinical Trials Registry Platform for registered intervention studies that may have examined barriers and facilitators to dietary adherence as secondary outcomes. This search will be conducted as part of our grey literature strategy to identify potentially relevant studies that may not have been published or may be in progress. If we identify relevant unpublished studies, we will contact the authors to inquire about the status of the study and reasons for non-publication. For published studies, we will compare the outcomes reported in the methods section with those presented in the results to identify any selective reporting of outcomes, contacting study authors for clarification if selective outcome reporting is suspected. If we conduct any meta-analyses, we will use funnel plots to visually assess potential publication bias for outcomes with data from ten or more studies and consider using statistical tests for funnel plot asymmetry, such as Egger’s test [29].

To assess the overall strength of evidence for our findings, we will use the Grading of Recommendations Assessment, Development, and Evaluation (GRADE) approach [30] for quantitative outcomes and the GRADE-CERQual (Confidence in the Evidence from Reviews of Qualitative research) approach for qualitative findings [31]. The GRADE approach assesses the quality of evidence across studies for specific outcomes based on five domains: risk of bias, inconsistency, indirectness, imprecision, and publication bias. The overall quality of evidence is categorized as high, moderate, low, or very low (Appendix 4 in the supplementary file). For qualitative findings, the GRADE-CERQual approach assesses confidence in evidence based on four components: methodological limitations, coherence, adequacy of data, and relevance. The overall confidence in each review finding is rated as high, moderate, low, or very low. We will present a summary of findings tables for both quantitative and qualitative evidence, including the GRADE and GRADE-CERQual assessments.

## Discussion

This systematic review aims to provide a comprehensive synthesis of the barriers and facilitators to dietary adherence in patients with chronic kidney disease, using the TDF to guide the categorization and interpretation of findings. By integrating evidence from both quantitative and qualitative studies, we expect to gain a nuanced understanding of the complex factors that influence patients’ ability and willingness to adhere to dietary recommendations. The findings of this review will have several important implications for clinical practice, intervention development, policy, patient empowerment, and research.

Knowledge of the multifaceted nature of dietary adherence in CKD, structured according to the TDF domains, can inform more personalized and effective approaches to dietary counseling and support by healthcare providers. The identified barriers and facilitators, mapped to the TDF domains, will serve as a foundation for developing targeted, theory-informed interventions to improve dietary adherence, such as educational programs, behavioral interventions, or technological solutions tailored to address specific barriers and leverage key facilitators. By highlighting systemic barriers to dietary adherence, such as access to dietitian services or food insecurity, this review may inform health policy decisions and resource allocation to better support CKD patients in managing their diet. Understanding patients’ perspectives and experiences, as captured by the TDF domains, can contribute to more patient-centered care approaches, empowering patients to actively participate in their dietary management. Finally, this review will identify gaps in the current literature and suggest priorities for future research in the field of dietary management in CKD.

Strengths of this review include the comprehensive search strategy, inclusion of both quantitative and qualitative studies, rigorous methodology, use of the TDF to guide the categorization and interpretation of findings, and broad scope. The broad search terms and inclusion of multiple databases will ensure a thorough capture of relevant literature. Including both quantitative and qualitative studies will provide a more complete picture of the factors influencing dietary adherence, combining statistical associations with in-depth patient experiences. The use of established guidelines (PRISMA-P) and tools for quality assessment and evidence grading will enhance the reliability and transparency of our findings. The application of the TDF will provide a structured approach to understanding the complex influences on dietary adherence and inform the development of theory-informed interventions. Furthermore, by including studies across all stages of CKD and various dietary components, we will be able to explore how barriers and facilitators may vary across the disease trajectory and different dietary recommendations.

However, there are also several limitations to consider. The diversity of study designs, populations, and outcomes may limit our ability to conduct meta-analyses and draw definitive conclusions about the relative importance of different factors. Despite our efforts to identify unpublished studies, there remains a risk of publication bias, particularly for studies finding no significant associations or barriers/facilitators. Additionally, as dietary guidelines for CKD patients have evolved, older studies may reflect outdated recommendations, potentially affecting the relevance of identified barriers and facilitators. We also acknowledge that the mapping of barriers and facilitators to TDF domains involves some degree of subjective interpretation. To minimize this limitation, two reviewers will independently map factors to TDF domains, with disagreements resolved through discussion and consultation with experts in behavior change theory if necessary.

## Conclusion

This systematic review protocol outlines a comprehensive approach to synthesizing the evidence on barriers and facilitators to dietary adherence in patients with chronic kidney disease, using the TDF to guide the categorization and interpretation of findings. By integrating quantitative and qualitative evidence, we aim to provide a nuanced understanding of the complex factors influencing dietary management in CKD. The findings of this review will inform clinical practice, intervention development, and future research directions, ultimately contributing to improved dietary management and outcomes for CKD patients. Through our rigorous methodology and diverse dissemination plans, we hope to maximize the impact of this review on patient care and research in the field of CKD dietary management.

## Supplementary Information

Supplementary Material 1: Appendix 1. Search Strategy for Different Databases. Appendix 2. Data Extraction Form. Appendix 3. Quality Assessment Tools. Appendix 4. Sample Summary of Findings Table (GRADE).

## Data Availability

All data generated or analyzed during this systematic review are included in this published article and its supplementary materials.
