# Supplementary material for: Barriers and facilitators to dietary adherence in adults with chronic kidney disease: a protocol for systematic review
Source: Syst Rev. 2026 Mar 26;15:151. doi: 10.1186/s13643-026-03080-6 (PMC13141286; doi:10.1186/s13643-026-03080-6)
Supplement: Supplementary file 1 — Supplementary Material 1: Appendix 1. Search Strategy for Different Databases. Appendix 2. Data Extraction Form. Appendix 3. Quality Assessment Tools. Appendix 4. Sample Summary of Findings Table (GRADE). [file 13643_2026_3080_MOESM1_ESM.docx]

Appendix A: Search Strategy for Different Databases

1. PubMed:

(("kidney"[MeSH Terms] OR "renal"[All Fields] OR "nephr*"[All Fields] OR "CKD"[All Fields]) AND ("diet*"[All Fields] OR "nutrition"[MeSH Terms] OR "nutritional"[All Fields] OR "food"[MeSH Terms] OR "eat*"[All Fields]) AND ("adherence"[All Fields] OR "compliance"[All Fields] OR "self-management"[MeSH Terms] OR "engag*"[All Fields]) AND ("barrier*"[All Fields] OR "facilitator*"[All Fields] OR "factor*"[All Fields] OR "determinant*"[All Fields] OR "challenge*"[All Fields] OR "motivator*"[All Fields] OR "cause*"[All Fields] OR "relationship*"[All Fields] OR "influence*"[All Fields] OR "obstacle*"[All Fields] OR "connection*"[All Fields] OR "qualitative*"[All Fields]))

2. Web of Science:

TS=((kidney OR renal OR nephr* OR CKD) AND (diet* OR nutrition OR nutritional OR food OR eat*) AND (adherence OR compliance OR self-management OR engag*) AND (barrier* OR facilitator* OR factor* OR determinant* OR challenge* OR motivator* OR cause* OR relationship* OR influence* OR obstacle* OR connection* OR qualitative*))

3. Embase:

('kidney'/exp OR renal OR nephr* OR 'ckd'/exp) AND (diet* OR 'nutrition'/exp OR nutritional OR 'food'/exp OR eat*) AND (adherence OR compliance OR 'self management'/exp OR engag*) AND (barrier* OR facilitator* OR factor* OR determinant* OR challenge* OR motivator* OR cause* OR relationship* OR influence* OR obstacle* OR connection* OR qualitative*)

4. CINAHL:

((MH "Kidney+") OR renal OR nephr* OR CKD) AND (diet* OR (MH "Nutrition+") OR nutritional OR (MH "Food+") OR eat*) AND (adherence OR compliance OR (MH "Self Care+") OR engag*) AND (barrier* OR facilitator* OR factor* OR determinant* OR challenge* OR motivator* OR cause* OR relationship* OR influence* OR obstacle* OR connection* OR qualitative*)

5. MEDLINE:

((exp Kidney/ OR renal OR nephr* OR CKD) AND (diet* OR exp Nutrition/ OR nutritional OR exp Food/ OR eat*) AND (adherence OR compliance OR exp Self Care/ OR engag*) AND (barrier* OR facilitator* OR factor* OR determinant* OR challenge* OR motivator* OR cause* OR relationship* OR influence* OR obstacle* OR connection* OR qualitative*))

6. The Cochrane Library:

((kidney OR renal OR nephr* OR CKD) AND (diet* OR nutrition OR nutritional OR food OR eat*) AND (adherence OR compliance OR self-management OR engag*) AND (barrier* OR facilitator* OR factor* OR determinant* OR challenge* OR motivator* OR cause* OR relationship* OR influence* OR obstacle* OR connection* OR qualitative*))

7. PsycINFO:

((DE "Kidney" OR renal OR nephr* OR CKD) AND (diet* OR DE "Nutrition" OR nutritional OR DE "Food" OR eat*) AND (adherence OR compliance OR DE "Self-Management" OR engag*) AND (barrier* OR facilitator* OR factor* OR determinant* OR challenge* OR motivator* OR cause* OR relationship* OR influence* OR obstacle* OR connection* OR qualitative*))

8. Scopus:

TITLE-ABS-KEY((kidney OR renal OR nephr* OR CKD) AND (diet* OR nutrition OR nutritional OR food OR eat*) AND (adherence OR compliance OR self-management OR engag*) AND (barrier* OR facilitator* OR factor* OR determinant* OR challenge* OR motivator* OR cause* OR relationship* OR influence* OR obstacle* OR connection* OR qualitative*))

Appendix B: Data Extraction Form

| Category | Item |
| --- | --- |
| Study Identification | - Author(s)  - Year of publication  - Title  - Journal |
| Study Characteristics | - Study design  - Country  - Setting (e.g., outpatient, inpatient)  - Sample size  - Duration of follow-up |
| Participant Characteristics | - Age (mean, SD, range)  - Gender distribution  - Ethnicity  - CKD stage  - Comorbidities  - Duration of CKD  - Renal replacement therapy status |
| Dietary Adherence | - Definition of adherence used  - Method of assessing adherence  - Specific dietary components assessed  - Reported adherence rates |
| Barriers to Adherence | - Identified barriers  - Method of identifying barriers  - Strength of association (if reported) |
| Facilitators of Adherence | - Identified facilitators  - Method of identifying facilitators  - Strength of association (if reported) |
| Additional Outcomes | - Patient-reported experiences  - Other relevant findings |
| Analysis Methods | - Statistical methods (quantitative studies)  - Qualitative analysis approach |
| Study Quality | - Risk of bias assessment  - Other quality indicators |
| Notes | - Any additional relevant information |

Appendix C: Quality Assessment Tools

Newcastle-Ottawa Scale (NOS) for non-randomized studies

| Selection | Max. stars |
| --- | --- |
| 1) Representativeness of the exposed cohort | * |
| 2) Selection of the non-exposed cohort | * |
| 3) Ascertainment of exposure | * |
| 4) Demonstration that outcome of interest was not present at start of study | * |

| Comparability | Max. stars |
| --- | --- |
| 1) Comparability of cohorts on the basis of the design or analysis | ** |

| Outcome | Max. stars |
| --- | --- |
| 1) Assessment of outcome | * |
| 2) Was follow-up long enough for outcomes to occur | * |
| 3) Adequacy of follow up of cohorts | * |

Critical Appraisal Skills Programme (CASP) Qualitative Checklist

| Section | Question |
| --- | --- |
| Screening | 1. Was there a clear statement of the aims of the research? |
|  | 2. Is a qualitative methodology appropriate? |
| Detailed questions | 3. Was the research design appropriate to address the aims of the research? |
|  | 4. Was the recruitment strategy appropriate to the aims of the research? |
|  | 5. Was the data collected in a way that addressed the research issue? |
|  | 6. Has the relationship between researcher and participants been adequately considered? |
|  | 7. Have ethical issues been taken into consideration? |
|  | 8. Was the data analysis sufficiently rigorous? |
|  | 9. Is there a clear statement of findings? |
| Value of the research | 10. How valuable is the research? |

Appendix D: Sample Summary of Findings Table (GRADE)

| Outcomes | Impact | № of participants (studies) | Certainty of the evidence (GRADE) |
| --- | --- | --- | --- |
| Barrier: Limited knowledge of dietary requirements | Patients with limited knowledge of dietary requirements were more likely to report poor adherence | 500 (5 observational studies) | ⨁⨁◯◯ LOW |
| Facilitator: Family support | Patients with strong family support reported better dietary adherence | 350 (3 observational studies) | ⨁⨁⨁◯ MODERATE |
| Barrier: Food cost and availability | Higher food costs and limited availability of appropriate foods were associated with poorer adherence | 600 (4 observational studies) | ⨁⨁◯◯ LOW |
| Facilitator: Regular dietitian support | Regular access to dietitian support was associated with improved dietary adherence | 450 (3 observational studies, 1 RCT) | ⨁⨁⨁◯ MODERATE |

GRADE Working Group grades of evidence High certainty: We are very confident that the true effect lies close to that of the estimate of the effect Moderate certainty: We are moderately confident in the effect estimate: The true effect is likely to be close to the estimate of the effect, but there is a possibility that it is substantially different Low certainty: Our confidence in the effect estimate is limited: The true effect may be substantially different from the estimate of the effect Very low certainty: We have very little confidence in the effect estimate: The true effect is likely to be substantially different from the estimate of effect
